# Spontaneous Splenic Rupture in Malaria Patients: Two Case Reports

**DOI:** 10.7759/cureus.20344

**Published:** 2021-12-11

**Authors:** Ahmed M Alani, Jouhar J Kolleri, Ahmad Al Ekeer, Zeinab Alsiddig A Ibrahim

**Affiliations:** 1 Clinical Imaging Department, Hamad Medical Corporation, Doha, QAT

**Keywords:** spontaneous splenic rupture, computed tomography, ultrasonography, plasmodium vivax, plasmodium falciparum, malaria

## Abstract

Atraumatic splenic rupture is a rare and life-threatening condition, if not diagnosed. We present two cases with a history of travel to endemic areas, who came to the emergency department with abdominal pain and were diagnosed to have spontaneous splenic rupture as a complication of severe malaria. Both patients were treated surgically by splenectomy. A high level of clinical suspicion is critical in every malaria patient presenting with abdominal pain, even if it is mild. Clinical imaging modalities like ultrasonography and computed tomography (CT) are crucial diagnostic tools in managing such patients.

## Introduction

Malaria is a mosquito-borne infection caused by parasites from the plasmodium group. There are five parasite species that cause malaria in humans. Plasmodium falciparum (P. falciparum) and Plasmodium vivax (P. vivax) are the most common organisms that can cause malaria in humans [1]. Malaria can lead to severe complications, such as shock, respiratory distress, severe anemia, multiple convulsions, and intra-abdominal organ inflammation [2]. Spontaneous rupture of the spleen is an extremely rare entity and occurs in up to an estimated 2% of cases [3]. We present two patients who came to the emergency department with abdominal pain and were diagnosed to have splenic rupture as a complication of malaria.

## Case presentation

Case 1

A 38-year-old gentleman with no past medical history presented to the emergency department with complaints of left upper quadrant abdominal pain for four days. The pain was severe, constant, and associated with nausea and vomiting. He had recent travel history to Ghana, normal bowel and bladder habits, and denied any history of trauma or fall. His vitals were as follows: temperature: 36.5 degrees Celsius, heart rate: 84 beats per minute, respiratory rate: 20 per minute, blood pressure: 113/76 mmHg, and oxygen saturation: 98%. On abdominal examination, there was marked tenderness in the left hypochondrium. Other systemic examinations were within normal limits.

**Figure 1:**
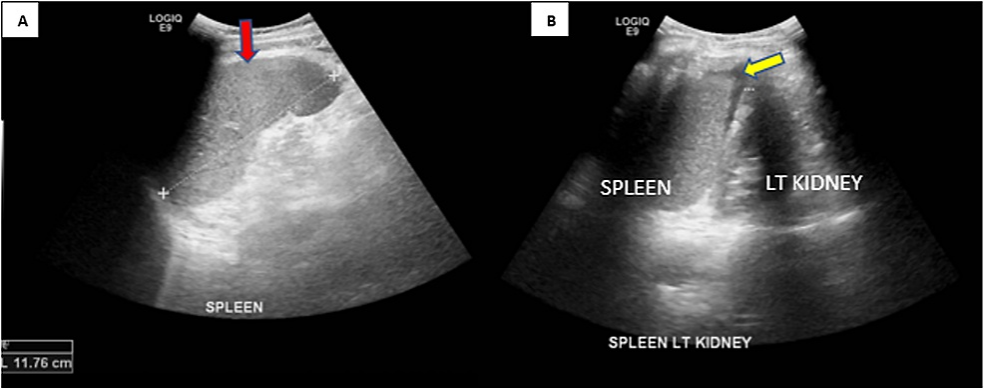
Ultrasound abdomen showing A) mildly enlarged spleen measuring 11.76 cm (red arrow) and B) trace of fluid in the splenorenal space (yellow arrow)

Laboratory examinations revealed high c-reactive protein levels and low platelet count. Malaria smear test was positive showing early trophozoite state (ring forms), suggestive of falciparum malaria. Ultrasound abdomen was done and showed mildly enlarged spleen, with no obvious focal lesion. A trace amount of fluid was seen in the splenorenal space (Figure 1). He was started on oral artemether-lumefantrine tablets 20 mg/120 mg, 4 tablets twice daily for three days.

**Figure 2:**
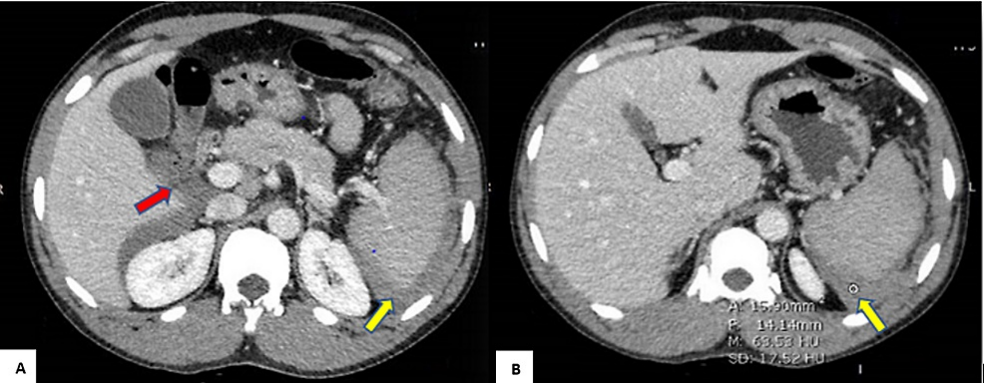
A) & B) computed tomography with intravenous contrast, axial cuts showing right upper abdomen hemoperitoneum (red arrow), and perisplenic hematoma (yellow arrows)

On the second day of admission, the patient was still having severe abdominal pain, despite taking regular analgesics. CT abdomen with contrast was performed and revealed perisplenic hyperdense collection or hematoma with suspicion of a distorted capsule at the inferior border, hemoperitoneum, and multiple mesenteric lymph nodes enlargement (Figure 2).

The patient was managed as a case of acute abdomen due to spontaneous rupture of the spleen and underwent emergency laparotomy and splenectomy. During the operation, splenic laceration, tear in the capsule, significant amount of clotted and non-clotted blood in both upper compartments, and moderate splenomegaly were detected. The patient was discharged on the sixth day after surgery. Influenza and pneumococcal vaccine were given on the day of surgery. On the fifth postoperative day, Haemophilus influenzae and meningococcal vaccines were given. The patient was followed up to 10 days postoperatively and was clinically stable.

Case 2

A 34-year-old gentleman, with no past medical history, presented to the emergency department with a history of left upper abdominal pain and fever for three days. He had a history of travel to Nepal recently. No history of nausea or vomiting was noted. His vitals were as follows: temperature 40.6 °C (oral), heart rate: 129 beats per minute, blood pressure: 92/50 mmHg, respiratory rate: 20 per minute, and oxygen saturation: 99%. On general examination, he was dehydrated. Local examination revealed mild tenderness in the left hypochondrium.

**Figure 3:**
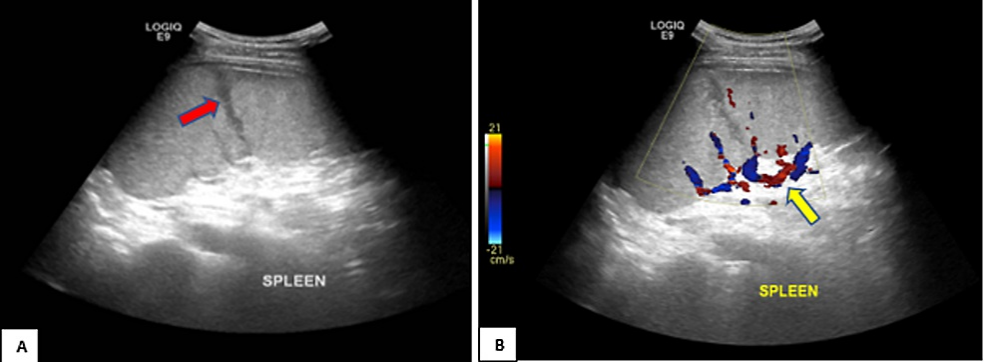
Ultrasound abdomen showing A) focal hypoechoic linear area in the spleen extending from the cortex (red arrow) and B) no vascularity on Doppler imaging (yellow arrow)

Intravenous normal saline was given, and laboratory workups showed high c-reactive protein. Malarial smear was positive for plasmodium vivax malaria. The patient was admitted to the critical care unit as a case of severe malaria. Ultrasound abdomen showed mild splenomegaly with hypoechoic linear echogenicity extending from the cortex and no vascularity on doppler examination that suggested splenic laceration (Figure 3).

**Figure 4:**
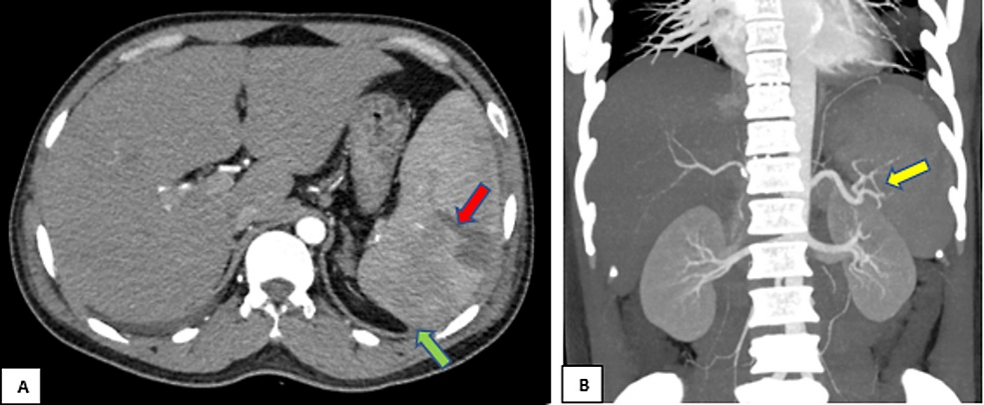
Computed tomography with intravenous contrast A) axial and B) coronal cuts showing a hypodense parenchymal laceration at the posterior aspect of the spleen (red arrow) with mild perisplenic hematoma (green arrow) and intact splenic artery (yellow arrow)

Conservative treatment was given initially with intravenous fluids and anti-malarial drugs artemether-lumefantrine 20 mg/120 mg tablets, four tablets, twice daily for three days, and primaquine 30 mg, once daily for 12 days, which showed initial improvement, but later he had a drop in hemoglobin along with hypotension and severe dehydration. CT abdomen with contrast was performed, and it showed features of splenic laceration of grade III with a mild volume of hemoperitoneum and preserved splenic vessels, giving an impression of spontaneous splenic rupture (Figure 4).

The patient underwent splenectomy and postoperative days were uneventful. Influenza and pneumococcal vaccines were given on the day of surgery. Meningococcal and Haemophilus influenza vaccines were given on the fourth postoperative day and discharged home on Day 7 with follow-up in the surgical outpatient clinic.

## Discussion

Malaria parasites have been around since the dawn of time. They most probably evolved in Africa along with humans, and mosquito fossils reveal that malaria vectors have been there for at least 30 million years. Humans are the sole vertebrate hosts for Plasmodium parasites and Anopheles mosquitoes are the vectors. This parasite specificity also suggests a long and adaptive association with humans [4].

In malaria, the precise mechanism of spleen rupture is unknown. The following three mechanisms are implicated in the process of splenic rupture: i) Increased intrasplenic tension owing to cellular hyperplasia and engorgement, ii) spleen compression by abdominal muscle during physiological activities like sneezing, coughing, defecation, etc., iii) vascular occlusion due to reticuloendothelial hyperplasia, culminating in thrombosis and infarction [3]. This causes interstitial and subcapsular hemorrhage, as well as capsule stripping, which leads to even more subcapsular hemorrhage. Finally, the swollen capsule breaks away, leading to splenic rupture [5].

Most cases of spontaneous splenic rupture linked with Plasmodium vivax occur during acute malaria illness. A main predisposing factor appears to be a lack of prior immunity to malaria [6]. Although spontaneous splenic rupture in malaria patients usually happens in acute infection with a grossly enlarged spleen, it may happen with a mildly enlarged spleen as in one of our cases. So, if the patient has malaria and presented with left upper quadrant pain, with no history of trauma, and shows normal findings or some free fluid around spleen, and in CT scan, there is mild pericapsular or subcapsular hyperdense fluid as in our cases, there should be high suspicion of spontaneous splenic rupture.

Conservative management with antimalarial drugs is the best option for patients who are hemodynamically stable. For children and adults with P. falciparum malaria, artemether-lumefantrine is the effective and safe treatment, whereas, for the dormant liver stage of P. vivax, primaquine is the treatment of choice [7-8]. Nonoperative management consists of observation for seven to 14 days in the hospital, strict bed rest, and administration of fluid and blood as needed [9]. To assess the healing of the ruptured spleen repeated ultrasonography or CT scan is recommended, which is done in two to three weeks. Splenectomy should be reserved for patients with uncontrollable bleeding, hemodynamically unstable, and patients with ongoing shock that is nonresponsive to resuscitation with fluids [10]. Embolization of the splenic artery can be performed in well-equipped facilities [1].

There is growing evidence that treatment of spontaneous rupture of the malarial spleen without splenectomy should be attempted in malaria-endemic areas [9]. In our cases, immediate splenectomy was the standard plan of management to prevent fatal complications and hypovolemic shock, which could happen because of intra-abdominal bleeding. The risk of sepsis caused by encapsulated bacteria is higher in splenectomized individuals. This group should get vaccinated against pneumococcal, meningococcal, and Haemophilus influenza B [11].

## Conclusions

Spontaneous splenic rupture in complicated malaria is extremely rare. There should be a high index of suspicion for spontaneous splenic rupture in a patient who is diagnosed to have malaria and presented with left upper quadrant pain, even without a history of trauma. Ultrasound and computed tomography will aid in early diagnosis to avoid the fatal complications of malaria. Perisplenic hematoma with hemoperitoneum should be managed with abdominal exploration. And nonoperative management is the best option if the patient is hemodynamically stable. Prophylactic measures should be taken by travelers to endemic areas.
